# King Edward's Hospital Fund for London

**Published:** 1905-12-16

**Authors:** 


### KING EDWARD'S HOSPITAL FUND FOR LONDON.

### DISTRIBUTION MEETING.

A meeting of the General Council of King Edward's Hospital Fund for London, for the purpose of awarding grants to the hospitals and convalescent homes for the present year, was held on the 8th instant, at 15 Portman Square.

The Duke of Fife, who is acting as president of the fund during the absence of the Prince of Wales, presided. Those present were: The Lord Mayor of London, the Governor of the Bank of England (Mr. A. F. Wallace), the President of the College of Surgeons (Mr. John Tweedy), the Chief Rabbi (the Rev. Dr. Adler), Sir Savile Crossley, M.P., Sir W. S. Church, Sir Joseph Dimsdale, M.P., Sir Henry Burdett, Sir John Aird, M.P., Sir William Collins, Sir John Craggs, Mr. Hugh Colin Smith, Mr. Julius Wernher, Mr. F. M. Fry, and Mr. J. Danvers Power. The minutes of the last meeting having been read and confirmed, letters of regret were reported from the following: The Lord Lieutenant of Middlesex (the Duke of Bedford), the Duke of Norfolk, Lord Stanley, M.P., Lord Iveagh, Lord Strathcona, Lord Rothschild, the Bishop of London, the Bishop of Southwark, Archbishop Bourne, Sir Trevor Lawrence, Mr. Thomas Burt, M.P., Mr. William Latham, K.C., the Rev. J. Guinness Rogers, D.D., the Rev. T. Bowman Stephenson, D.D., Mr. E. A. Hambro, Mr. J. Pierpont Morgan, jun., Mr. Albert G. Sandeman, and Mr. Edgar Speyer.

Sir John Craggs reported that the amount received for general purposes by the fund to December 2 amounted, after payment of expenses, to £93,865.

Mr. Hugh C. Smith (chairman) presented the report of the Executive Committee as follows: The Executive Committee recommend this year the distribution of £100,000 to the London Hospitals, making, with the £1,000 entrusted to the fund by the London Parochial Charities, for the Convalescent Homes, a total distribution of £101,000. It will be seen from the accounts when they are made up that, including legacies and other amounts not specifically allocated to capital by testators or donors, the income for the year was in excess of £100,000. The committee, however, felt that, inasmuch as the amount received in legacies during this year was much in excess of the average, and as in previous years sums have been taken from capital to make up the amounts distributed, it would not be prudent to distribute more than the amount recommended. The Executive Committee have adopted the reports and recommendations of the Hospitals Distribution Committee and the Convalescent Homes Distribution Committee.

Report of the Distribution Committee.

The chairman of the Hospitals Distribution Committee (Sir William Church) presented the report as follows:—

"1. The committee have the pleasure to report that the Executive Committee, with the approval of his Royal Highness the President, placed in their hands this year the sum of £100,000 for distribution among the London hospitals, as against £79,000 in 1904.

"2. The number of hospitals applying for grants this year remains at 106, six having fallen out and six new applications having been received.

"3. The committee desire to reiterate their strong conviction of the undesirability of opening new hospitals without the fullest consideration both as to their necessity and as to the means of maintaining them.

"4. In reference to the City Orthopædic Hospital, the committee, having been informed that there is now a prospect of the hospital joining in the amalgamation of the National and Royal Orthopædic Hospitals, have recommended a grant subject to the arrangements made being approved by the Executive Committee.

"5. The committee learn with satisfaction that negotiations for the amalgamation of the various Throat, Nose, and Ear Hospitals are in progress.

"6. With regard to applications from newly-established hospitals for grants from the fund, the committee recommend the adoption of the practice followed by the Hospital Sunday Fund, which requires that under ordinary circumstances a hospital should have been maintained in existence for three complete years before becoming eligible to participate.

"7. The committee are glad to note that St. George's Hospital and the Westminster Hospital have made arrangements to transfer the teaching of the preliminary and intermediate subjects of medical education to University centres, as recommended in last year's report.

"8. A sum of £7,500 has already been contributed by the King's Fund to the special fund for the removal of King's College Hospital to South London; a further grant of £4,000, making in all £11,500, is now recommended in order to further this most desirable undertaking.

"9. The question of fire prevention, to which a paragraph was devoted in last year's report, has received the careful consideration of the Executive Committee, who, it is understood, are awaiting the decision of the London County Council with regard to the inspection of public buildings before reporting to the General Council.

"10. The committee have this year made two special grants in order to enable hospitals to accomplish specific objects which might be prejudiced by delay. In the case of the Great Northern Central Hospital an adjoining piece of freehold land, now for sale, should be at once secured in the interests of the charity; and the committee have recommended that the whole of the purchase price, £1,600, should be granted. In the case of the West Ham Hospital, which is about to enter into possession of an adjoining building recently acquired and much needed, the committee think that a sum of £2,500 to cover the cost of furniture and fittings might be granted at once in order that the benefits of the extension may be immediately available for this poor district. The fact that both these hospitals are this year receiving more than would have been the case under ordinary circumstances, and that parts of the grants recommended must be regarded in the nature of advances, will of course be taken into consideration in the future.

"11. The attention of the committee has been called to the considerable grants made in past years towards building schemes, which in some instances have not yet been carried out. They would suggest that the Executive Committee should consider whether in future similar grants might not be retained by the fund until the money is actually required and credited, together with the accrued interest, to the hospitals to which they were made.

"12. The committee have, as heretofore, divided the grants into annual grants and donations. They recommend that in future grants made by the fund should not be divided in this way. The fund has now attained a permanent income of £50,000 per annum, which is more than sufficient to enable it to pay all the annual grants which it has hitherto made. There is, therefore, no longer any question as to its being able in future years to carry out its undertakings. Moreover, it must be remembered that many hospitals which only receive donations are as much in need of yearly help as those receiving annual grants. The fact has also been brought to the notice of the committee that in some cases where grants have been allocated for the reopening of beds the beds reopened have not permanently added to the accommodation of the hospitals, inasmuch as other wards have been subsequently closed. In other instances the hospitals to which grants have been made for the reopening of beds no longer require the assistance of the fund for that purpose. It seems, therefore, that the proper time has come for making the proposed change.

"13. The reports of the visitors have, as usual, received the careful consideration of the committee and have been of material assistance in enabling them to apportion their awards. It is desirable to point out that circumspection with regard to suggestions should still be maintained, lest any let fall by the visitors on the occasion of their visits should be interpreted, as has more than once been the case, as official recommendations, and afterwards quoted as the sanction for expenditure which it is sought to defray, in whole or in part, by grants from the fund.

"14. In addition to their published remarks the committee have followed their usual practice in recommending that various suggestions should be made privately to many of the hospitals. They would add that they have taken into special consideration several cases where the expenditure appears to be unreasonably high, and also others where efforts are being made to reduce excessive expenditure."

### List of Grants to the Hospitals.

Sir Savile Crossley read the awards as follows :—

The Distribution Committee desire to draw attention to the fact that the absence of a grant does not necessarily imply dissatisfaction. In the following list the committee's "Remarks" follow the amounts of the grants in each case :—

Alexandra Hospital for Children.—£500 donation.

Belgrave Hospital.—£1,000 donation; £500 to debt. The committee view with satisfaction the removal to South London.

Blackheath and Charlton Cottage Hospital.—£50 donation.

Bolingbroke Hospital.—£2,500 donation; to building fund.

British Lying-in Hospital.—£100 donation.

Cancer Hospital.—No grants. An excellent institution.

Central London Ophthalmic Hospital.—£150 donation.

Central London Throat and Ear Hospital.—£300 donation. In view of the negotiations in progress for the amalgamation of the Throat and Ear Hospitals, the Distribution Committee would regard with grave dissatisfaction any steps towards rebuilding at present.

Charing Cross Hospital.—£2,500 donation; £1,000 to building debt.

Chelsea Hospital for Women.—£350 donation.

Cheyne Hospital.—£100 donation; in consideration of the fact that curable cases are admitted.

City of London Hospital for Diseases of the Chest, Victoria Park, E.—£1,500 annual; £750 donation.

City of London Lying-in Hospital.—£500 donation; to building.

City Orthopædic Hospital.—£250 donation; subject to the arrangements to be made for amalgamation being approved by the Executive Committee.

Clapham Maternity Hospital.—£100 donation.

Dreadnought Hospital.—£500 annual; £1,500 donation.

East End Mothers' Home.—£200 donation; £50 to service lift.

East London Hospital for Children.—£1,700 donation; £700 towards improvements. The committee view with satisfaction the economical management.

Eltham and Mottingham Cottage Hospital.—£25 donation.

Evelina Hospital.—£100 donation. The committee will be prepared to give substantial help towards the reconstruction of the out-patient department when the work has been commenced.

French Hospital.—£200 donation.

Friedenheim Hospital.—£50 donation.

General Lying-in Hospital.—£150 donation.

German Hospital.—£200 donation.

Gordon Fistula Hospital.—£50 donation.

Great Northern Central Hospital.—£750 annual; £2,350 donation; special grant, £1,600, to provide freehold land (*vide* paragraph 10 in Report). The committee view with satisfaction the economical management.

Grosvenor Hospital for Women and Children.—£100 donation.

Guy's Hospital.—£5,000 annual; £3,000 donation; £3,000 to debt. The committee view with satisfaction the economical management.

Hampstead General Hospital.—£2,000 donation; for building.

Home and Infirmary for Sick Children.—£150 donation.

Home for Mothers and Babies and Training School for District Midwives.—No grants; *vide* Report, paragraph 6.

Hospital for Consumption.—£1,000 donation.

Hospital for Diseases of the Throat.—£100 donation. The committee would view with satisfaction an amalgamation of the Throat and Ear Hospitals.

Hospital for Epilepsy and Paralysis.—£450 donation.

Hospital for Invalid Gentlewomen.—£200 donation.

Hospital for Sick Children.—£500 annual; £1,000 donation.

Hospital for Women.—£3,100 donation; £2,000 for building.

Hospital of St. John and St. Elizabeth.—£500 annual.

Infants' Hospital.—No grants. *Vide Report*, paragraph 6.

Invalid Asylum.—No grants. The committee consider that this asylum is outside the scope of the fund.

Italian Hospital.—£500 annual.

Kensington Dispensary and Children's Hospital.—£25 donation.

King's College Hospital.—£1,000 annual; £5,000 donation; £4,000 to the fund for the removal of the hospital to South London, which the committee view with great satisfaction.

London Fever Hospital.—£200 donation.

London Homœopathic Hospital.—£400 donation.

London Hospital.—£5,000 annual; £3,000 donation.

London Lock Hospital (Female).—£1,500 donation; £1,000 towards improving nursing accommodation.

London Temperance Hospital.—£1,000 donation; to out-patient department.

London Throat Hospital.—£50 donation. The committee would view with satisfaction an amalgamation of the Throat and Ear Hospitals.

Medical Mission Hospital (formerly entered as Cottage Hospital, Canning Town), 538, 540 Barking Road, E.—£200 donation; £100 to building.

Memorial Cottage Hospital (Mildmay Park).—£50 donation.

Metropolitan Hospital.—£500 annual; £500 donation.

Middlesex Hospital.—£1,000 annual; £1,250 donation; and Middlesex Hospital (Cancer Charity), no grants. The committee in making their grant have, as hitherto, considered the cancer charity in conjunction with the hospital, and therefore do not now separate it.

Mildmay Mission Hospital.—£250 donation.

Miller Hospital, Greenwich.—£1,250 donation; £500 to extension.

Mount Vernon Hospital for Consumption (formerly entered as North London Hospital for Consumption). —£1,000 annual; £1,500 donation. The committee view with satisfaction the economical management.

National Anti-Vivisection Hospital.—No grants.

National Dental Hospital.—£25 donation; to debt.

National Hospital for the Paralysed and Epileptic.—£750 annual; £1,250 donation.

National Orthopædic Hospital.—£400 donation. This grant is given pending the amalgamation with the Royal Orthopædic Hospital, which the committee view with great satisfaction, and for which a special sum is set aside.

New Hospital for Women.—£500 donation.

North-Eastern Hospital for Children.—£500 annual; £500 donation; £500 to reduce loan.

North-West London Hospital.—£750 donation.

Norwood Cottage Hospital.—£25 donation.

Oxygen Hospital.—No grants.

Paddington Green Children's Hospital.—£600 donation; £200 towards debt.

Passmore Edwards' Acton Cottage Hospital.—No grants. The committee are pleased to see that the hospital is not in need of help from the fund this year.

Passmore Edwards' Hospital, East Ham.—£150 donation.

Passmore Edwards' Hospital for Willesden.—£25 donation.

Passmore Edwards' Cottage Hospital, Wood Green.—No grants.

Poplar Hospital.—£500 donation.

Queen Charlotte's Lying-in Hospital.—£500 donation.

Queen's Jubilee Hospital.—No grants. The committee feel unable to recommend a grant to this hospital until they are satisfied that the recent changes in the management have secured efficient administration.

Royal Dental Hospital of London (formerly entered as Dental Hospital).—£500 donation; to debt.

Royal Ear Hospital.—£500 donation; to building debt. The committee would view with satisfaction an amalgamation of the Throat and Ear Hospitals.

Royal Eye Hospital.—£350 donation.

Royal Free Hospital.—£750 annual; £2,250 donation; £1,500 to improvements.

Royal Hospital for Diseases of the Chest, City Road.—£400 donation; the £500 given in 1903 to open-air sanatorium, not since constructed, to be released and applied towards installation of the electric light.

Royal London Ophthalmic Hospital.—£2,000 annual; £1,500 donation. The committee consider this hospital should receive more support from the public.

Royal Orthopædic Hospital.—£200 donation. This grant is given pending the amalgamation with the National Orthopædic Hospital, which the committee view with great satisfaction, and for which a special sum is set aside.

Royal Waterloo Hospital for Children and Women.—£1,500 donation; £1,000 to building debt.

Royal Westminster Ophthalmic Hospital.—£200 donation; to debt.

St. George's Hospital.—£2,000 donation. The committee view with satisfaction the efforts being made to reduce expenses, and the transference of instruction in the preliminary and intermediate subjects of medical education to University centres.

St. John's Hospital for Diseases of the Skin.—£50 donation.

St. John's Hospital, Morden Hill, Lewisham, S.E.—£150 donation; £50 towards improvements.

St. Luke's House.—£25 donation.

St. Mark's Hospital.—£75 donation.

St. Mary's Children's Hospital, Plaistow.—£1,500 donation; £1,000 to building.

St. Mary's Hospital, Paddington.—£1,000 annual; £2,300 donation; £1,300 to building debt.

St. Monica's Hospital.—£100 donation.

St. Peter's Hospital for Stone.—£75 donation; towards lift.

St. Saviour's Hospital, Osnaburgh Street.—£200 donation.

St. Thomas's Hospital.—No grants. The committee are not satisfied that the hospital is in need of present assistance from the fund.

Samaritan Free Hospital.—£500 annual; £300 donation; £300 to out-patient department.

Santa Claus Home.—£25 donation. In consideration of the fact that curable cases are admitted.

South Wimbledon and Merton Cottage Hospital.—No grants.

Tottenham Hospital.—£1,000 annual; £2,000 donation; £2,000 to building. The committee view with satisfaction the economical management.

University College Hospital.—£1,000 annual; £1,000 donation.

Victoria Hospital for Children.—£1,000 donation; £500 to building debt.

West-end Hospital for Diseases of the Nervous System.—£150 donation.

Western Ophthalmic Hospital.—£150 donation; to debt.

West Ham Hospital.—£300 annual; £2,500 donation. Special grant, £2,500, to provide the furniture and fittings for the new wards. (*Vide* Report, paragraph 10.)

West London Hospital.—£1,500 annual; £1,000 donation; £1,000 for debts. The committee view with satisfaction the economical management.

Westminster Hospital.—£750 annual; £2,000 donation; £2,000 to discharge existing debts. The committee view with satisfaction the transference of instruction in the preliminary and intermediate subjects of medical education to University centres.

Woolwich and Plumstead Cottage Hospital.—£25 donation.

Total.—Annual grants, £27,800; donations, £72,200. Total grants to hospitals for the year 1905, £100,000.

### Grants to Convalescent Institutions.

In the absence of Mr. William Latham, K.C., chairman of the Convalescent Homes Committee, Mr. F. M. Fry read the report as follows: "Owing to the continued liberality of the trustees of the London Parochial Charities the committee have again £1,000 to distribute. The committee are bound to consider the qualifications of applicants in respect, first, to their connection with London, and, secondly, of their coming fairly within the definition of convalescent homes. They have therefore given the preference to institutions directly connected with London hospitals." The following awards are recommended :—

Alexandra Hospital, Convalescent Home at Painswick.—£25.

All Saints' Convalescent Hospital, Eastbourne.—£50.

Charing Cross Hospital, Convalescent Home at Limpsfield, Surrey.—£50.

East London Hospital for Children, Convalescent Home at Bognor.—£100.

Hospital for Sick Children, Convalescent Branch, Cromwell House, Highgate, N.—£50.

King's College Hospital, Convalescent Home at Hemel Hempstead.—£100.

London Hospital, Convalescent Home at Tankerton, near Whitstable.—£50.

Metropolitan Hospital, Convalescent Home at Cranbrook. —£25.

Metropolitan Convalescent Institution, 32 Sackville Street, Piccadilly, W.—£200.

Middlesex Hospital, Convalescent Home at Clacton-on-Sea.—£100.

Mildmay Mission Hospital, Convalescent Home at Hendon.—£25.

Mrs. Gladstone's Free Convalescent Home for the Poor, Ravensbury House, Mitcham, Surrey.—£25.

Paddington Green Children's Hospital, Convalescent Home at Slough.—£100.

Queen Charlotte's Lying-in Hospital, Convalescent Home at 20 Victoria Road, Kilburn, N.W.—£25.

Victoria Hospital for Children, Convalescent Home at Broadstairs.—£75.

Total grants to Convalescent Homes, £1,000.

The Duke of Fife, in moving the adoption of the report of the Executive Committee, said : I shall not venture to occupy your time by making any lengthy remarks of my own, as I consider myself as merely the mouthpiece of our President, whose absence we naturally regret, although we rejoice to know it is caused by his having again undertaken a great Imperial mission. On the work of this fund, and the admirable results which have been achieved, I have nothing but the warmest congratulations to offer to you, and to all those who have so kindly and patriotically devoted themselves to the work. If this fund had done nothing more than create a greater interest in the London hospitals and improve their organisation it would have earned the profound gratitude of the whole community. But it has done far more. Thanks to the magnificent generosity of some of its supporters, it has rendered very material assistance to the great institutions which it was founded to benefit. The good work of the League of Mercy has enlisted the sympathies and support of a class which it would otherwise have been difficult to reach, and I am sure that you will agree with me when I say that all honour is due to the ladies and gentlemen who have worked so enthusiastically and successfully in its cause. Much as has been done of late years for the London hospitals, I feel that I could not occupy my present position to-day without repeating what I have said publicly on other occasions, that their financial condition still leaves a great deal to be desired, and I fear that this will remain the case until the London public will take a more adequate view of its duty towards its hospitals.

### A Letter from the Prince of Wales.

In spite of the existence of this and other funds, we cannot disguise from ourselves the fact that the great majority of the London hospitals are only kept alive by somewhat unsound methods of finance and continuous sensational appeals, and now, with your permission, I will read a letter which I have received from the Prince of Wales :—

"It is a matter of sincere regret to me that my absence in the East prevents me from being present at this year's distribution meeting of the council of King Edward's Hospital Fund for London. This is the first occasion upon which I have been unable to preside since my appointment by the King to the presidency.

"Please assure the meeting of my ever keen interest in the work and welfare of the fund.

"Although I only know the results of the past year up to November, enough is already shown to prove, as I trust all will agree, that they are most satisfactory and encouraging.

"We have now secured a permanent income from investments amounting to £50,000 a year. I hope that, as the work of the fund becomes better known, there will be an equally gratifying increase in the annual subscriptions.

"I am glad to see that there are indications of the hospitals having already benefited to some extent from the statistical reports which have been issued to them by the fund. I look forward with much interest to the results which will be shown in the next report, as it covers the working of a complete year since the first statistical report was available.

"I have learnt with satisfaction that the recommendations of the Medical Schools Committee have already been adopted by some of the London hospitals. Our only object has been, while strictly adhering to the original aim and object of the fund, to assist by the best advice procurable, in furthering the movement for the establishment of a more systematic and co-ordinated scheme of medical education in the metropolis. Such a result would, we hope, be beneficial to the medical profession, of which we are so justly proud, and to whose co-operation in our work we owe so much.

"I have to thank Mr. J. G. Griffiths for a most valuable report, made at my request, on the existing system of hospital accounts. I trust that in his recommendations may be found a solution of the various difficulties which have hitherto confronted the hospital authorities in this important branch of their management.

"My best thanks are due to the honorary officers and committees of the fund for their untiring and valuable services, and to each and all of those who serve on the General Council.

"Let me add, in conclusion, I am confident that the administration of the fund is safe in the hands of those to whom it is entrusted, and I am greatly indebted to you for acting in my behalf during my absence."

The Lord Mayor seconded the adoption of the report, which was carried.

It was proposed by Mr. H. C. Smith, seconded by Sir Joseph Dimsdale, M.P., and resolved, that cheques for the grants should be posted on December 14.

The Governor of the Bank of England (Mr. A. F. Wallace) proposed, and Sir John Aird, M.P., seconded, a vote of thanks to the President, which was carried unanimously, and which the Duke of Fife acknowledged.

